# Clinical Decision-Making Case: Pediatric Sexually Transmitted Infections and Consent

**DOI:** 10.21980/J8.52335

**Published:** 2025-12-31

**Authors:** Emily Drone, Andrew Shedd, Leslie Rodriguez, Chinmay Patel

**Affiliations:** * John Peter Smith Hospital, Department of Emergency Medicine, Fort Worth, TX; ^ Baylor Scott and White All Saints Medical Center, Department of Emergency Medicine, Fort Worth, TX; † Baylor Scott and White All Saints Medical Center, Department of Graduate Medical Education, Fort Worth, TX

## Abstract

**Audience:**

This clinical decision-making case for the Certifying Board Exam is designed for emergency medicine residents at all training levels (PGY1 through PGY4).

**Introduction:**

Navigating consent for pediatric patients in the emergency department (ED) presents unique ethical and legal challenges. Physicians must understand parental consent requirements and the relevant exceptions that apply in the ED. Studies indicate that residents may lack confidence or knowledge in handling complex or nuanced consent scenarios, particularly regarding adolescents.^1^ We aimed to develop a structured educational intervention to address this gap. This clinical decision-making case aims to improve resident competency and comfort in managing these situations.

**Educational Objectives:**

By the end of this case the learner will be able to: 1) demonstrate competency with the new ABEM Certifying Exam Clinical Decision-Making Case format, 2) manage a simulated pediatric care encounter that requires navigating the details of pediatric consent, 3) explain common exceptions to requiring parental consent in emergency situations according to established guidelines as well as state and local laws, 4) report increased comfort managing ethical dilemmas related to pediatric consent in the ED.

**Educational Methods:**

This educational activity utilizes the new Clinical Decision-Making Case format for the American Board of Emergency Medicine Certifying Board Exam. This method simulates realistic ED encounters where residents must gather information, apply ethical and legal principles, and make decisions regarding pediatric consent under time constraints, similar to the new structure used in the board certification process for emergency medicine physicians. Additionally, a short presentation accompanied the debrief of this session to highlight the relevant clinical learning points.

**Research Methods:**

We administered pre- and post-intervention surveys assessing self-perceived comfort (using Likert scales) and objective knowledge (using multiple-choice questions) regarding pediatric consent, as well as comparing audience vs. participants experience. This study was approved by the Baylor Research Institute Institutional Review Board, approval number 025-322. Informed consent was obtained from all participants electronically.

**Results:**

Thirteen EM residents (PGY1–PGY3) participated in this activity. In the presurvey, only 30.8% of residents reported to be somewhat or very comfortable, while in the post survey, 100% reported to be somewhat or very comfortable with pediatric consenting. When asked to evaluate the learning value of the case, 76.9% selected very valuable and 15.4% selected valuable.

**Discussion:**

This clinical decision-making case provides a standardized, active learning method to address emergency medicine residency training regarding pediatric consent, which has previously been identified as an area of difficulty for EM trainees. 1 The format allows for assessment of not just knowledge, but also application, communication, and ethical reasoning. Providing specific, constructive feedback immediately following the session is crucial for maximizing educational benefit.

**Topics:**

Clinical decision-making case, board certification, pediatrics, ethics, legal.

## USER GUIDE

**Table:** 

List of Resources:	
Abstract	166
User Guide	168
For Examiner Only	170
Certifying Exam Assessment	176
Stimulus	178
Debriefing and Evaluation Pearls	185

**Learner Audience:**

Interns, Junior Residents, Senior Residents

**Time Required for Implementation:**

Case: Clinical Decision-Making cases are 15 minutes as directed by American Board of Emergency Medicine (ABEM).

Debriefing: 15 minutes

**Recommended number of learners per instructor:**

one active learner per instructor, with a variable number of observers as is standard practice in your institution.

**Topics:**

Structure clinical encounter, board certification, pediatrics, ethics, legal.

**Objectives:**

By the end of this case, learners will be able to:

1. Demonstrate competency with the new ABEM board certification structured interview format.
2. Manage a simulated pediatric care encounter that requires navigating the details of pediatric consent.
3. Explain common exceptions to requiring parental consent in emergency situations according to established guidelines and state law.
4. Report increased comfort managing ethical dilemmas related to pediatric consent in the ED.

### Linked objectives, methods and results

This Clinical Decision-Making Case for the Certifying Board Exam was designed to teach the legal and ethical nuances of pediatric consent through active resident learning. The case is structured to have the learner simulate real-life emergency medicine practice, progressing through the patient care steps consistent with the new ABEM board certification format (Objective 1). As learners manage the simulated encounter, they must navigate the specific details of providing care and addressing confidentiality for a minor patient presenting without a guardian (Objective 2). The case prompts are designed to directly assess the learner’s ability to explain the common exceptions to requiring parental consent in emergency situations (Objective 3). By successfully managing this challenging encounter in a protected learning environment, which is followed by a faculty-led debrief, the intervention is designed to meet the final objective of increasing learner comfort in managing ethical dilemmas related to pediatric consent in the ED (Objective 4).

### Recommended pre-reading for instructor

- Katz AL, Webb SA. Informed consent in decision-making in pediatric practice. *Pediatrics*. 2016;138(2):e20161485. doi:10.1542/peds.2016-1485
- Sirbaugh P. Consent for emergency medical services for children and adolescents. *Pediatrics*. 2011;128(2):427–433. doi:10.1542/peds.2011-1166

### Results and tips for successful implementation

**Figure 1:**
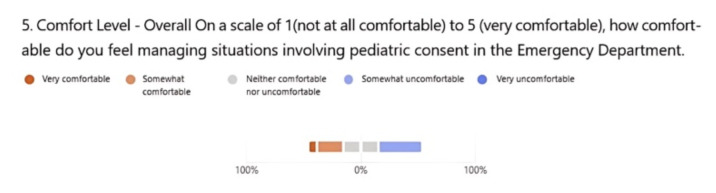
Pre-Survey Resident Comfort Levels

**Figure 2:**
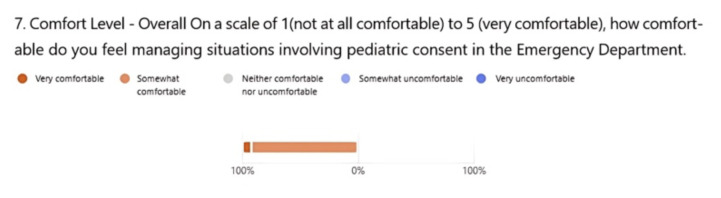
Post Survey Resident Comfort Levels

For our institution, we ran this case during our pediatric block in resident conference. We had one resident run the case with the faculty proctor with the rest of the residents running the case as observers. After the case, the proctor delivered a brief lecture on the topic, engaging the audience and connecting the lecture to the case. This is a standard format for our program, and as such we perceived no barriers to learning between the participant vs. observers. We measured our success with both a pre-and-post survey of the residents (both observers and participants) and noted a significant improvement in perceived comfort with the topic of pediatric consent. In the presurvey, 30.8% of residents reported to be somewhat or very comfortable with pediatric consenting (Figure 1). After completing the clinical decision-making case, 100% reported to be somewhat or very comfortable with pediatric consent (Figure 2). Based on faculty availability, we believe this could also be done in a small group setting to optimize engagement. Overall, our residents noted improved comfort on a topic that has been both locally and nationally recognized as a common knowledge gap.

## Figures and Tables

**Table:** 

**I. History**		Yes	No
**1a**	Elicit that patient is pregnant		
**1b**	Patient denies abdominal or pelvic pain		
**2**	Confidentiality despite minor patient		
**3**	No parental consent required due to complaint related to sexual health/pregnancy		
**II. Physical Examination**			
**4a**	Pelvic exam to evaluate for cervical discharge, CMT, adnexal tenderness		
**4b**	Focused abdominal exam to evaluate for tenderness		
**5**	To evaluate for CMT or adnexal/uterine tenderness		
**III. Differential Diagnosis**			
**6a**	Pelvic inflammatory disease/cervicitis		
**6b**	STI/gonorrhea/chlamydia		
**6c**	Third appropriate diagnosis such as trichomonas, bacterial vaginosis, candida vaginitis, TOA, etc.		
**IV. Diagnostic Studies**			
**7a**	Wet prep		
**7b**	Urinalysis		
**7c**	Panel for STI (Gonorrhea and Chlamydia)		
**8a**	***Rationale for PROMPT 8 #1		
**8b**	***Rationale for PROMPT 8 #2		
**V. Treatment and Other Actions**			
**9**	Treatment with ceftriaxone and azithromycin		
**10**	Empiric treatment for gonorrhea and chlamydia		
**VI. Final Diagnosis**			
**11**	Cervicitis in pregnancy		
**VIII. Disposition**			
**12**	Discharge		
**13**	No admission criteria such as TOA/sepsis		
**IX. Transitions of Care**			
**14**	Explaining diagnosis to the patient, follow-up with OB/gyn and offering resources		
**15**	Sexual health/pregnancy related complaints confidential despite being a minor		

**Table:** 

**Candidate Task Sheet**	
**#1**	**Emergency Department Admitting Form**
**#2**	**Historical Information**
**#3**	**Physical Exam Findings**
**#4**	**Diagnostic Studies and Results**

**Table:** Clinical Decision-Making Task Sheet

**CASE PARAMETERS**
This is a 15-minute case You will interact with two examiners. This is an interview style without role playing; you should simply reply to the questions asked. You may be interrupted to move you through the case; this is not a reflection of your performance.
**PATIENT INFORMATION**
16-year-old female with vaginal discharge
**VITAL SIGNS**
BP: 104/68 P: 98 R: 16 T: 98.6° F 02Sat: 100% on RA
**TASK STATEMENT**
Your tasks are as follows: List pertinent elements of a focused history and physical exam Develop an appropriate differential and/or provisional diagnosis Select and interpret appropriate studies Articulate appropriate patient management including discharge instructions

**STIMULUS 1:** Emergency Department Admitting Form

**Patient Information**	
**Patient Name**	Jane Smith
**Age**	16
**Gender**	Female
**Method of Arrival**	Private vehicle
**General Appearance/History of Present Illness**	Patient presents with abnormal vaginal discharge for about a week. She also reports she is approximately 10 weeks pregnant but hasn’t told her parents yet. She is no acute distress.
**Vital Signs on ED Arrival**	BP: 104/68 P: 98 R: 16 T: 98.6° F O2 sat: 100% on RA

**STIMULUS 2:** Historical Information

**History of Present Illness/Description of Event**	
Patient denies any abdominal or pelvic pain. Patient reports she is sexually active with her boyfriend without use of contraception. Last menstrual period was approximately 8 weeks ago. Patient has had positive home pregnancy tests. Patient reports she lives at home with her mother and father and feels safe but is worried because she has not told them about the pregnancy test yet.	
**History**	
**Past Medical History**	No known medical problems
**Past Surgical History**	None
**Medications**	None
**Allergies**	No known drug allergies
**Social**	Patient lives with her mother and father who are not aware of the pregnancy. Occasional sexual encounters with similar aged males with inconsistent barrier protection.

**STIMULUS 3:** Physical Exam Findings

**Physical Examination**	
**General Appearance**	Awake, alert, no acute distress
**Dermatologic**	Warm, dry, no rashes
**HEENT**	Atraumatic, normocephalic, moist mucous membranes
**Neck**	Supple, no cervical lymphadenopathy
**Respiratory**	Clear to auscultation bilaterally, no respiratory distress
**Cardiac**	Normal rate and rhythm, no murmurs, rubs, or gallops
**Abdominal**	Soft, nontender, no distension, no rebound or guarding
**Extremities**	Good pulses, no edema or cyanosis
**Neurologic**	Alert & oriented x3, cranial nerves II–XII intact, strength 5/5 all extremities, normal sensation
**Pelvic**	Normal external female genitalia. No bleeding noted. Cervical os is closed. The cervix is erythematous with copious yellow discharge. There is no CMT, adnexal or uterine tenderness on bimanual examination. No masses are felt.
**Psych**	Mildly anxious, normal affect, no suicidal ideation

**STIMULUS 4:** Diagnostic Studies and Results

**Wet Prep**	WBC	Moderate
	Yeast	Absent
	Clue Cells	None
	Trichomonas	Absent

**Urinalysis.**	Appearance	Clear
	Color	Yellow
	Glucose	Negative
	Ketones	Negative
	Sp Gravity	1.020
	Blood	Negative
	pH	6.0
	Protein	Negative
	Nitrite	Negative
	Leukocyte	25
	WBC	6–10
	RBC	0–2
	Squamous Cells	0–5
	Bacteria	Absent

**Urine HCG**	Positive	

**Transvaginal US**	CLINICAL INDICATION: Rule out ectopic pregnancy. Confirm intrauterine pregnancy and viability. Estimate gestational age. Patient reports vaginal discharge. Last menstrual period 8 weeks prior. COMPARISON: None. TECHNIQUE: Real-time transvaginal grayscale and color Doppler ultrasound imaging of the pelvis was performed. Images were obtained in multiple planes. IMPRESSION: Single live intrauterine pregnancy. Estimated gestational age based on Crown-Rump Length (CRL) is 8 weeks and 0 days, consistent with the provided last menstrual period. Closed Cervical Os No sonographic evidence of ectopic pregnancy. No adnexal masses or significant free fluid.	

## CERTIFYING EXAM ASSESSMENT

*Clinical Decision-Making Case: Pediatric Sexually Transmitted Infections and Consent*

Learner: _________________________________________

**Summative and formative comments:**

## DEBRIEFING AND EVALUATION PEARLS

### Clinical Decision-Making Case: Pediatric Sexually Transmitted Infections and Consent^7^,^8^

**Emergency Medical Conditions (EMC):** In situations where a pediatric patient presents to the Emergency Department with a life-threatening condition or an emergency medical condition where treatment cannot be safely delayed, providers must proceed with stabilization. Consent is implied in these scenarios when a legal guardian is unavailable, and physicians should document that the treatment was initiated for an EMC to prevent harm to the child.

**Condition-Specific Consent Exceptions:** Once an EMC has been excluded, minors generally require parental consent, but exceptions exist for specific diagnoses that can include sexually transmitted infections (STIs), pregnancy-related care, sexual assault, and chemical addiction.

**The “Mature Minor” and Emancipation:** Educators should distinguish between specific condition exceptions and the care of an emancipated minor; a minor is considered legally emancipated (a “mature minor”) if they are on active-duty military service, married, or living apart from guardians and financially independent. These minors have the legal capacity to consent to all their medical care, not just protected services.

**State-Specific Legal Variability:** It is critical to note that consent and emancipations laws are not federally uniform and vary significantly by jurisdiction, particularly regarding “mature minor” status and privacy laws for adolescents. For example, criteria for emancipation or consent for contraception found in one region may not apply in other regions. Providers must actively consult their specific state statutes and institutional legal guides to ensure compliance rather than relying on general rules. We recommend the facilitator become familiar with their laws specific to their region prior to the session.
